# Analysis of prevalence, years lived with disability, and trends of anemia burden and main causes in China

**DOI:** 10.3389/fpubh.2025.1564756

**Published:** 2025-05-29

**Authors:** Pengfei Wang, Xue Cheng, Qiya Guo, Li He, Lahong Ju, Xiaoli Xu, Weiyi Gong, Shujuan Li, Liyun Zhao, Hongyun Fang

**Affiliations:** National Institute for Nutrition and Health, Chinese Center for Disease Control and Prevention, Beijing, China

**Keywords:** anemia, older adults, disease burden, trend, main causes

## Abstract

**Background:**

China is facing a rapidly aging population. In 2020, the number of people aged 60 and above was 264 million, accounting for 18.7% of the total population. Therefore, addressing the health issues of older adults is of great importance. This study aimed to analyze trends of anemia burden and its underlying causes in older adults aged 60 and above.

**Methods:**

Three indicators were used to evaluate the disease burden of anemia: prevalence, Years Lived with Disability (YLDs), and Years Lived with Disability rate (YLD rate). Data on anemia prevalence were obtained from the 2002 Chinese Nutrition and Health Survey, the 2010–2013 Chinese Nutrition and Health Surveillance, and the 2015 China Adults Chronic Diseases and Nutrition Surveillance. A multi-stage stratified cluster random sampling method was adopted in these surveys. YLDs, YLD rate (per 100,000 population), and causes of anemia were sourced from the Global Burden of Disease 2021 (GBD 2021). The Cochran-Armitage trend test was used to test the trends between different ages, genders, and living areas.

**Results:**

The prevalence and YLD rate of anemia among older adults aged 60 and above exhibited a notable decreasing trend across various demographic factors. Anemia prevalence was higher in rural areas, western areas, and southern China. The YLD rate of females was higher than that of males, except for adults over 85. Both prevalence and YLD rates increased with age. Dietary iron deficiency was the leading cause of anemia.

**Conclusion:**

While the disease burden of anemia has shown a decreasing trend, significant age, and regional disparities persist. Anemia among older adults, particularly in rural and western regions, remains a major health concern. Special attention is needed to address dietary iron deficiency as a key factor contributing to anemia.

## 1 Introduction

Population aging is a global phenomenon, that has significant implications for China as well. According to the results of the Seven National Population Census, there were 264 million people aged 60 and over in 2020, accounting for 18.7% of China’s total population (1). By 2040, this number is expected to reach 402 million (accounting for 28% of the total population), according to WHO estimates (2). The physiological functions of older adults begin to decline, which results in changes to the performance of different organ systems (3), and the immune function of older adults is also in decline (4). Anemia not only seriously affects the health of older adults, but also increases the social burden (5, 6). It associations with cognitive impairment, physical measures (such as mean handgrip, relative handgrip, and usual gait speed), and late recall in older adults (7). Anemia also increases the risk of heart failure, coronary heart disease, and mortality in older adults (8–11).

The burden of anemia is assessed using Years Lived with Disability (YLDs) since it does not directly cause mortality, making Years of Life Lost (YLLs) inapplicable (12). From 1990 to 2021, although the prevalence and YLD rate (per 100,000 population) of anemia decreased, the global total number of cases and YLDs of anemia still increased by 0.42 million and 3.4 million, respectively. At the national level, Mali, Zambia, Togo, and Senegal had a prevalence of anemia above 50% in 2021 (13). In China, the prevalence of anemia among adults was 8.3% in 2015, a decrease of 2.1 percentage points compared with 2012 (14). However, due to the physiological decline associated with aging and the increasing older adults population in China, anemia among older adults remains a significant concern that cannot be overlooked (1, 4).

Anemia can be caused by a variety of factors, but its root cause is an imbalance between erythrocyte production and loss at the biological level. This includes ineffective or deficient erythropoiesis and excessive erythrocyte loss (15). Ineffective or deficient erythropoiesis can result from nutritional deficiencies and inflammation, while excessive erythrocyte loss is typically caused by hemolysis and blood loss (12, 16–19). Understanding the underlying causes of anemia in older adults is crucial for developing effective strategies to prevent and control this health issue in older adults.

While most anemia research in China has focused on pregnant women and children, this study shifts its attention to older adults, a demographic that has been largely overlooked in existing literature. By integrating data from three national cross-sectional surveys and the Global Burden of Disease (GBD) database, this study provides a comprehensive and comparative analysis of the anemia burden among older adults. By examining 35 causes of anemia, this study offers a comprehensive assessment of the diverse causes among older adults, identifying key causes to inform evidence-based interventions and policy-making. In this study, the distribution of anemia in different population characteristics and underlying causes among older adults in China were analyzed, which was critical for developing interventions suitable for the actual situation of China and reducing the burden of anemia.

## 2 Methods

### 2.1 Study design and participants

The data on the prevalence of anemia were obtained from the 2002 Chinese Nutrition and Health Survey (2002 CNHS), the 2010–2013 Chinese Nutrition and Health Surveillance (2010–2013 CNHS), and the 2015 China Adults Chronic Diseases and Nutrition Surveillance (2015 CACDNS). Multi-stage stratified cluster random sampling method was adopted in these three surveys or surveillance. Nationally representative samples covering 31 provinces, autonomous regions, and municipalities directly under the Central Government were obtained. The specific sampling methods (14, 20, 21) were shown in Table 1. The ethical approval numbers were No. 2013-018 and 201519-B, respectively. Informed consent was signed by all participants before the survey/surveillance.

**Table 1:** Multistage cluster sampling in three-round surveillance.

	2002	2010–2013	2015
The first stage	6 regions by economic development level, 22 counties/districts from each type of region	4 tiers: large urban, mid-sized cities, rural (typical/disadvantaged), 150 surveillance sites	302 survey sites, 3 communes/subdistricts from each site
The second stage	3 communes/subdistricts from each county/district	6 neighborhood committees from each surveillance site	2 communities/villages from each township/subdistrict
The third stage	2 communities/villages from each commune/subdistrict	about 1,000 people from 450 households in six neighborhood committees	1 village/residential group
The fourth stage	90 households	_	45 households

The YLDs, YLD rate (per 100,000 population), and causes of anemia were obtained from the Global Burden of Disease 2021 (GBD 2021). The GBD 2021 estimated burden of 371 diseases and injuries using 100,983 data sources (22). The YLDs and YLD rate of anemia for older adults aged 60 years and above were sourced from the GBD 2021 database for anemia disease burden analysis. A total of 35 detailed causes of anemia were selected for analysis of the underlying causes of anemia. Since this data was obtained from public databases, ethical review and informed consent were not required.

### 2.2 Definition of anemia

In accordance with the WHO criteria, anemia was defined as a hemoglobin concentration of <130 g/L in men aged 15 years and above, and <120 g/L in non-pregnant women aged 15 years and above (23). The impact of altitude on hemoglobin concentration was adjusted using the Equation 1 (13). Participants were restricted to permanent residents (residency ≥6 months within the 12 months prior to the survey). This criterion ensured that participants had sufficient time to physiologically adapt to the local altitude, thereby minimizing the confounding effects of short-term population mobility.


(1) $$\begin{array}{l} \mathrm{\Delta Hb}=-0.32\times (\text{elevation in meters}\times 0.0033)\\ +0.22\times {\left(\text{elevation in meters}\times 0.0033\right)}^{2} \end{array}$$

ΔHb: hemoglobin values requiring adjustment at various altitudes (g/L).

Elevation in meters: altitude of each survey point (m).

### 2.3 Data collection and measurements

The basic information in the survey or surveillance, including gender, age, living area, and other relevant details, was gathered through face-to-face interviews conducted by the China Center for Disease Control and Prevention (China CDC). Fasting venous blood samples were collected and analyzed using the ferrocyanide method. A rigorous quality control program was implemented, covering standardized protocols for questionnaires, training, equipment, reagent handling, and data entry.

### 2.4 Assessment of other variables

To assess the associations between age and anemia, all individuals were divided into six distinct age groups: 60–64 years, 65–69 years, 70–74 years, 75–79 years, 80–84 years, and > = 85 years. The residential areas were categorized into two groups based on urban and rural distinctions, and classified into three regional groups: the eastern region, which includes cities and provinces such as Beijing, Tianjin, Liaoning, Shandong, Hebei, Jiangsu, Zhejiang, Shanghai, Fujian, Guangdong, and Hainan; the central region, comprising Jilin, Heilongjiang, Shanxi, Henan, Anhui, Jiangxi, Hunan, and Hubei; and the western region, which encompasses Inner Mongolia, Ningxia, Gansu, Qinghai, Xinjiang, Tibet, Guizhou, Chongqing, Sichuan, Yunnan, and Guangxi. China is divided into southern and northern regions by the Qinling-Huaihe Line.

### 2.5 Statistical analysis

In order to improve the representativeness of the sample to the population, the sample data were adjusted by the post-stratification weighting method. And adjusted survey or monitoring results in 2002, 2010–2013, and 2015 based on national census results in 2000, 2010 and 2020, respectively. The calculation of post-stratification weighting (Weight) for each layer was shown in Equation 2 (14).


(2) $$\begin{array}{l} \text{Weight}=\text{Population}\;\mathrm{at}\;a\;\text{certain level}\\ ÷\text{The}\;\mathrm{sum}\;\text{of the sample weights}\;\mathrm{at}\;\text{that level} \end{array}$$

SAS 9.4 software was used for basic information description and statistical analysis. R software (Version 4.3.1) was used for plotting and result visualization. Categorical data were presented as numbers (percentages). The Weighted Chi-square test was used to analyze whether there were differences between the basic characteristics. The Weighted Cochran–Armitage trend test was used to assess trends across different characteristics. Statistical significance was determined based on two-sided *p*-values, with *p*-value less than 0.05 considered statistically significant.

## 3 Results

### 3.1 Basic characteristics of subjects

A total of 26,247, 32,820, and 48,553 individuals aged 60 and above were included in the 2002 CNHS, the 2010–2013 CNHS, and the 2015 CACDNS, respectively. In 2002, the male-to-female ratio was 1:1.01; the male-to-female ratio was 1:1.11 in 2010–2013; in 2015, the male-to-female ratio in this study was 1:0.99. There were statistically significant differences in age groups, places of residence, region, and north–south distribution of population in different years (*p* < 0.0001). Table 2 provides a detailed overview of the survey participants’ demographic characteristics in 2002, 2010–2013, and 2015.

**Table 2:** Demographic characteristics of older adults aged 60 and above in China in 2002, 2010–2013, and 2015 [n, %].

Characteristics	2002	2010–2013	2015	*χ* ^2^	*p*
Gender					
Male	13,069 (49.79)	15,564 (47.42)	24,354 (50.16)	1.0	0.6158
Female	13,178 (50.21)	17,256 (52.58)	24,199 (49.84)		
Age groups					
60–64	9,362 (35.67)	12,394 (37.76)	19,360 (39.87)	38.7	<0.0001
65–69	8,173 (31.14)	8,683 (26.46)	13,871 (28.57)		
70–74	5,151 (19.63)	6,262 (19.08)	8,096 (16.67)		
75–79	2,377 (9.06)	3,648 (11.12)	4,577 (9.43)		
80–84	917 (3.49)	1,391 (4.42)	1977 (4.07)		
> = 85	267 (1.02)	442 (1.35)	672 (1.38)		
Places of residence					
Urban	11,966 (45.59)	17,387 (52.98)	20,702 (42.64)	190.0	<0.0001
Rural	14,281 (54.41)	15,433 (47.02)	27,851 (57.36)		
Region					
Eastern Region	11,921 (45.42)	13,284 (40.48)	18,810 (38.74)	98.1	<0.0001
Central Region	6,568 (25.02)	9,930 (30.26)	14,748 (30.38)		
Western Region	7,758 (29.56)	9,606 (29.27)	14,995 (30.88)		
North/South					
Northern China	12,596 (47.99)	14,916 (47.76)	19,652 (40.48)	383.5	<0.0001
Southern China	13,651 (52.01)	17,904 (54.55)	28,901 (59.52)		
Total	26,247	32,820	48,553		

Weighted Chi-square Tests was applied.

### 3.2 Trends in prevalence of anemia

As shown in Table 3, in 2002, 2010–2013, and 2015, the prevalence of anemia among older adults demonstrated a notable decreasing trend across various demographic factors. This decline was consistent across genders, with both males and females experiencing reductions in anemia rates. Furthermore, the trend was evident across various age groups, suggesting an overall improvement in anemia prevalence among older adults. This decreasing trend was also observed in different places of residence, regions, and the north–south divide. The anemia prevalence in rural areas was higher than that in urban areas, and southern China compared to northern China, across all 3 years (*p* was less than 0.05). And in 2002 and 2015 the prevalence of anemia in western areas was higher than in eastern and central areas (*p* was less than 0.05). The prevalence of anemia increased with age, with the prevalence in the > = 85 age group being twice that of the 60–64 age group (*p* < 0.05).

**Table 3:** Prevalence of anemia in older adults aged 60 and above in 2002, 2010–2013, and 2015 [95%CI].

Characteristics	2002	2010–2013	2015	*Z*	*p*
Gender					
Male	22.7 (21.2, 24.3)	13.9 (11.8, 15.9)	9.2 (7.8, 10.5)	1279.7	<0.0001
Female	23.2 (21.4, 25.1)	13.5 (11.7, 15.2)	9.1 (7.6, 10.5)	1301.3	<0.0001
Age groups					
60–64	19.3 (17.8, 20.9)	10.7 (9.1, 12.2)	6.8 (5.7, 7.9)	1031.1	<0.0001
65–69	21.2 (19.6, 22.9)	12.1 (10.3, 13.1)	7.9 (6.8, 9.1)	899.3	<0.0001
70–74	23.5 (21.5, 25.5)	14.2 (12.1, 16.2)	8.9 (7.3, 10.5)	871.9	<0.0001
75–79	27.8 (25.2, 30.4)	16.5 (14.0, 18.9)	12.2 (10.1, 14.3)	609.3	<0.0001
80–84	30.3 (26.2, 34.4)	22.0 (18.2, 25.8)	14.9 (12.3, 17.4)	514.7	<0.0001
> = 85	39.3 (32.0, 46.6)	21.1 (15.6, 26.5)	15.5 (12.7, 18.4)	412.4	<0.0001
Places of residence					
Urban	17.4 (16.1, 18.6)	12.6 (11.3, 13.8)	7.4 (6.6, 8.2)	1167.8	<0.0001
Rural	25.9 (24.7, 27.1)	14.5 (13.2, 15.9)	10.5 (9.5, 11.5)	1374.5	<0.0001
Region					
Eastern Region	19.0 (16.5, 21.6)	13.7 (10.9, 16.6)	8.1 (5.7, 10.5)	1190.0	<0.0001
Central Region	23.8 (20.8, 26.8)	14.1 (10.5, 17.7)	9.4 (7.5, 11.4)	977.2	<0.0001
Western Region	28.2 (25.6, 30.8)	13.1 (10.1, 16.1)	10.0 (7.4, 12.6)	989.7	<0.0001
North/South					
Northern China	16.3 (14.8, 17.7)	10.2 (8.8, 11.5)	6.6 (5.4, 7.8)	988.2	<0.0001
Southern China	29.0 (27.2, 30.8)	16.5 (14.5, 18.4)	10.7 (9.2, 12.3)	1591.0	<0.0001

### 3.3 Trends in YLDs and YLD rate of anemia

**Figure 1:**
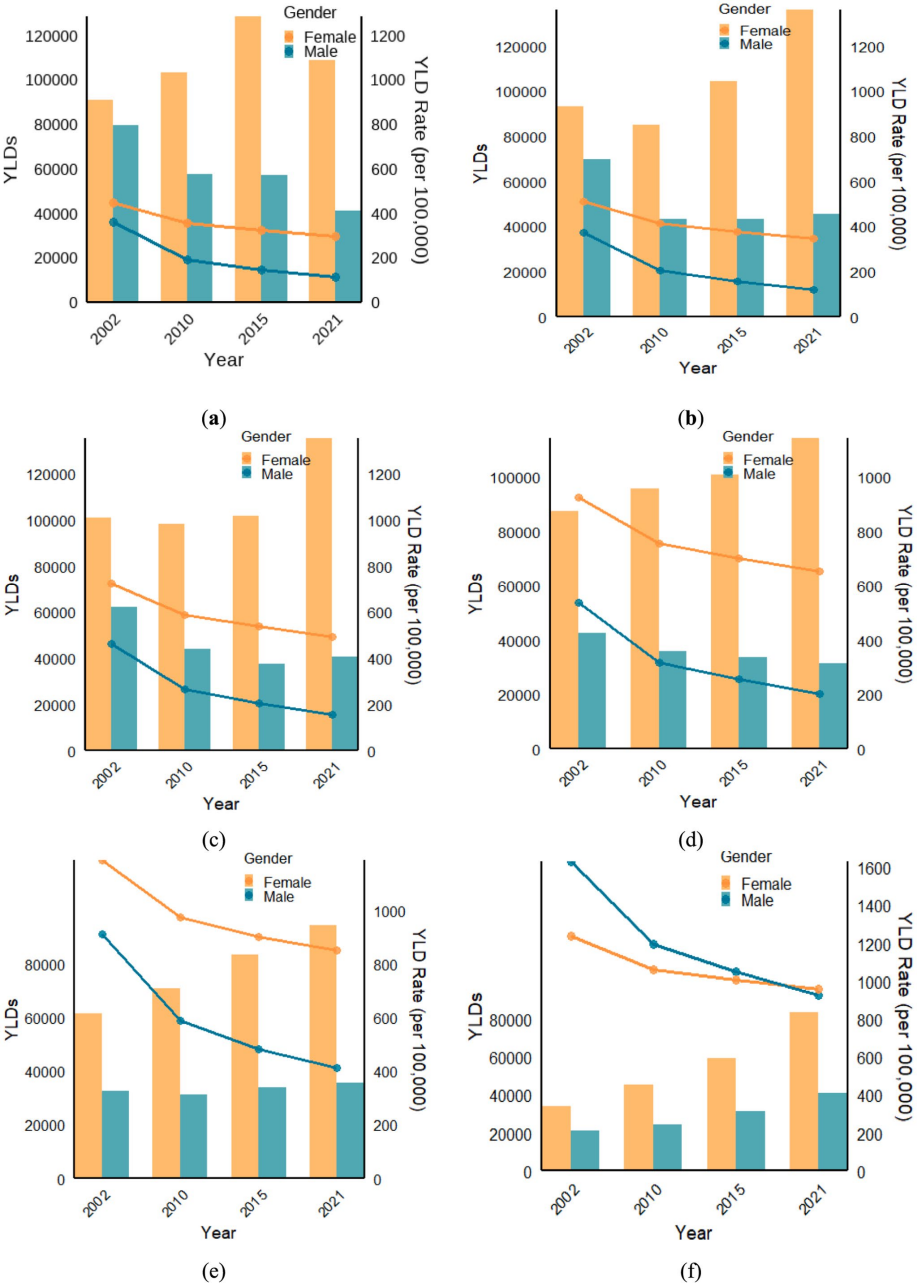
In 2002, 2010, 2015, and 2021, YLDs and YLDs Rate (per 100,000) in **(a)** the 60–64 age group; **(b)** the 65–69 age group; **(c)** the 70–74 age group; **(d)** the 75–79 age group; **(e)** the 80–84 age group; **(f)** the > = 85 age group.

As shown in Figure 1, in males, the YLDs for anemia showed a decreasing trend in the 60–64, 65–69, and 75–79 age groups, a decline followed by an increase in the 70–74 and 80–84 age groups, and an increasing trend in the 85 and older age group from 2002 to 2021. In females, the YLDs for anemia initially increased and then decreased in the 60–64 age group, decreased and then increased in the 65–69 and 70–74 age groups, and showed an increasing trend in the 75–79, 80–84, and 85 and older age groups from 2002–2021.

From 2002 to 2021, YLD rates (per 100,000) in males and females showed a downward trend. In the 60–64, 65–69, 70–74, 75–79, and 80–84 age groups, the YLD rates of anemia in females were higher than that in males. However, for individuals aged 85 and older, the YLD rates (per 100,000) for males were higher than for females in 2002, 2010, and 2015. In 2021, the YLD rates for females (960.6 per 100,000) exceeded that of males (927.5 per 100,000).

**Figure 2:**
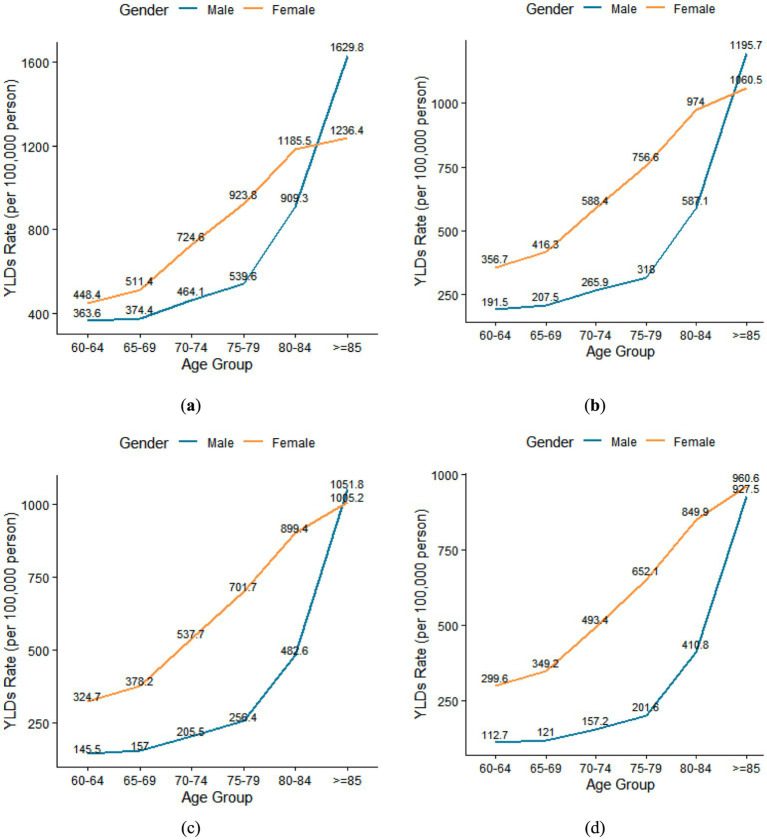
**(a)** Trends in YLDs Rate (per 100,000) of anemia with age in 2002; **(b)** Trends in YLDs Rate (per 100,000) of anemia with age in 2010; **(c)** Trends in YLDs Rate (per 100,000) of anemia with age in 2015; **(d)** Trends in YLDs Rate (per 100,000) of anemia with age in 2021.

In Figure 2, every year, the YLD rate of anemia showed an increasing trend with age in both males and females. In 2002, 2015, 2010, and 2021, the disease burden of anemia in males was 4.5, 6.2, 7.2, and 8.2 times higher, respectively, in the > = 85-year-old age group compared to the 60-year-old age group. The YLDs rate for males in the > = 85-year-old group was 1629.8 per 100,000 in 2002, 1195.7 in 2010, 1051.8 in 2015, and 927.5 in 2021.

Similarly, for females, the disease burden of anemia was 2.8, 3.0, 3.1, and 3.2 times higher in the > = 85-year-old age group compared to the 60-year-old age group in 2002, 2015, 2010, and 2021. The YLDs rate for females in the > = 85-year-old group was 1236.4 per 100,000 in 2002, 1060.5 in 2010, 1005.2 in 2015, and 960.6 in 2021.

Among older adults aged 60–84, the YLD rate of females was higher than that of males in 2002, 2010, 2015, and 2021. However, among older adults aged 85 and older, the YLD rate of males was higher than that of females in 2002, 2010, and 2015 (Figure 2).

### 3.4 The cause distribution of anemia burden in China in 2021

**Figure 3:**
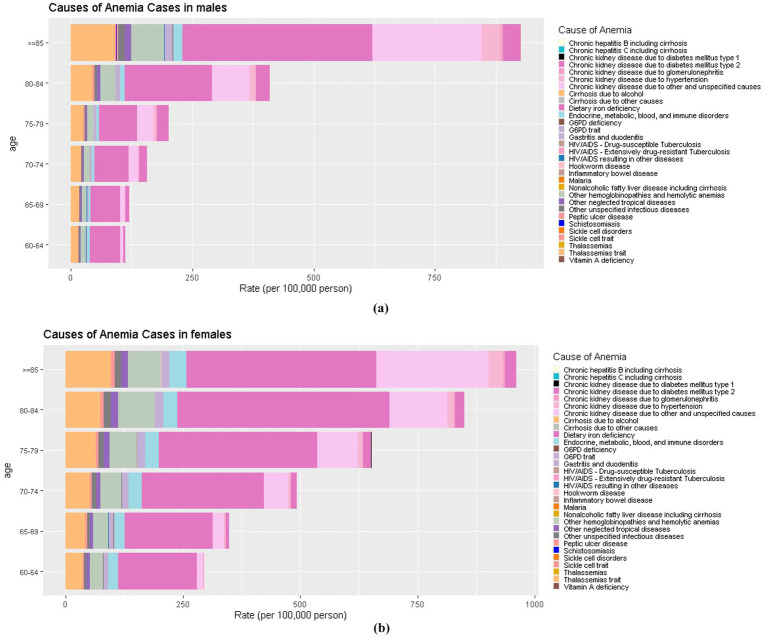
**(a)** The cause distribution of anemia burden in China in males; **(b)** The cause distribution of anemia burden in China in females.

In Figure 3 based on the 35 causes in the GBD 2021, in China, dietary iron deficiency was the leading cause of anemia across all age groups in both males and females. However, some variations were found in the distribution of anemia causes across different age groups and genders. In the 60–64 age group and 65–69 age group, the most common causes of anemia were dietary iron deficiency, thalassemias trait, and other hemoglobinopathies and hemolytic anemias. In the 70–74 age group, 75–79 age group, males of 80–84 age group, and > = 85 age group, the leading causes of anemia YLDs in China in 2021 were dietary iron deficiency, thalassemias trait, and chronic kidney disease due to other and unspecified causes. These three causes accounted for about 70% of all anemia cases. The main causes of anemia in females aged 80–85 were dietary iron deficiency, chronic kidney disease due to other and unspecified causes, and other hemoglobinopathies and hemolytic anemias, which account for 76.9% of all anemia causes.

## 4 Discussion

This study analyzed the temporal trend of the prevalence of anemia in older adults aged 60 years and above in 2002, 2010–2013, and 2015, as well as the YLDs and YLD rates of anemia. Furthermore, the causes of anemia were also investigated in this study. From 2002 to 2015, the prevalence of anemia among older adults aged 60 and above, showed a decreasing trend. Additionally, from 2002 to 2021, based on data collected in 2002, 2010, 2015, and 2021, the YLD rates caused by anemia exhibited a downward trend over time. However, age differences and regional differences in the burden of anemia cannot be ignored.

The prevalence of anemia among older adults showed a decreasing trend in both males and females, with a continuous downward trend across all age groups in China. From 1990 to 2020, the prevalence of anemia in males, females, and the overall population exhibits a significant negative correlation with the years (24). A related study also showed that the prevalence of anemia in older adults decreased from 30. 61%to 18. 15% (*χ*^2^ = 21723.90, *p* < 0.001), with the declining trend gradually slowing down from 2012 to 2018 (25). Females of childbearing age had a high prevalence of anemia due to pregnancy or menstrual bleeding, which has received widespread attention (26, 27). In older adults, a higher prevalence of anemia in males has been observed in the U.S. This higher anemia risk was partially explained by different dietary habits and lifestyle factors between males and females. Moreover, some cancers (such as oropharyngeal, and laryngeal) have a more significant impact on males (28–31). However, our study found no significant gender difference in anemia prevalence in China.

The prevalence of anemia among older adults showed a decreasing trend in different places of residence and regions, consistent with a related study (32). This may be because aging has become an important and pressing issue concerning national development and the well-being of the population. The health of the older adults has received significant attention. The State Council has issued a series of documents, including the “14th Five-Year Plan for National Aging Development and Older adults Care Service System” and the “Healthy China 2030 Plan” (33, 34). At the same time, the new management model based on “Internet+” healthcare platforms, using the Internet and telemedicine, has effectively integrated home, community, and medical institutions, meeting the health needs of older adults and reducing the incidence of anemia (35).

The study identified significant geographical disparities in anemia prevalence in China, characterized by higher rates in rural areas compared to urban areas, southern versus northern regions, and western versus to central-eastern areas. These differences were linked to socioeconomic, dietary, and environmental-genetic factors. Socioeconomic disparities were particularly evident in rural and western regions, where had poorer overall socioeconomic conditions, medical resources and health literacy (36–39). Additionally, although the urbanization rate in China rose 47.3% from 1978 to 2022, the dietary structures in urban and rural had significant differences. Compared to rural areas, the dietary structures in urban were closer to the recommendations of Dietary Guidelines for Chinese Residents 2022 (40). Dietary patterns in the south, with higher consumption of rice and vegetables, may affect iron absorption (41). The subtropical climate of southern regions fosters higher rates of soil-transmitted helminth infections, which induce chronic blood loss and exacerbate iron deficiency (42). Genetic predispositions, such as the higher prevalence of thalassemia traits in provinces like Guangdong and Guangxi, may impair hemoglobin synthesis and increase anemia risk (43). Therefore, enhancing socioeconomic conditions and ensuring the equitable distribution of medical resources could significantly help decrease the prevalence of anemia.

The YLD rates associated with anemia have shown a downward trend over time. However, there has been an increase in YLDs among both females and males in the 80–84 and ≥85 age groups. This increase may be linked to advancements in healthcare, which have led to longer life expectancies within the older adults population. Additionally, factors such as the decline in physical function and the high prevalence of underlying diseases have extended the duration of living with chronic conditions, which may contribute to a gradual increase in the burden of anemia-related diseases over the years (24).

The YLDs and YLD rates were higher in females than males, except for the YLD rate of > = 85 age group. One reason may be that although the prevalence of multiple cancer types was higher among males, the mortality rate was also higher, resulting in a shorter survival period for males compared to females (30). Females may be particularly vulnerable to food insecurity within households and might have limited access to iron-rich foods. Additionally, they were often less likely to receive health screenings and care due to domestic responsibilities, lack of autonomy, or prioritizing the needs of other family members (44–46). In females, the incidence of obesity, type 2 diabetes, and cardiovascular diseases (CVD) significantly increases after menopause (47). Obesity is accompanied by several disturbances at the endothelial, hormonal, and inflammatory levels. These disturbances induce the activation of several mechanisms that contribute to the anemic state (48). It has been reported that patients with type 2 diabetes are twice as likely to have anemia compared to individuals without type 2 diabetes (49). A related study showed that a higher genetically predicted risk for heart failure (HF), coronary artery disease (CAD), and ischemic stroke (AIS) was significantly positively associated with anemia risk (8). The usage rate of certain medications is higher in females than in males among older adults. For instance, one study found that 78% of females used bisphosphonates for the treatment of osteoporosis (50). There is an association between bisphosphonates and the risk of non-serious gastrointestinal adverse events (*RR* = 1.16, *CI*: 1.00–1.36), such as gastric pain, nausea, and vomiting (51). The prolonged occurrence of these adverse reactions may lead to a decrease in appetite and inadequate intake, thereby affecting the absorption of essential nutrients related to red blood cell production, such as iron, vitamin B12, and folate. Therefore, it is essential to implement targeted measures to address the gender inequalities that contribute to anemia.

The prevalence and YLD rate of anemia consistently increased with age, although there were variations between years. The prevalence of anemia in the 60–64 age group compared to the > = 85 age group, increased by 20.0, 10.4, and 8.7% in 2002, 2010, and 2015, respectively. The rate of increase gradually decreased. In 2021, the YLD rate was 3.2 times higher in the 85-year-old age group compared to the 60-year-old age group in females and was 8.2 times higher in males. This was consistent with related studies (28, 52). China is experiencing population aging, which indicates that anemia among older adults is a common health concern for us.

In this study, we found that dietary iron deficiency was the leading cause of anemia across all age groups in both genders. Thalassemias trait, other hemoglobinopathies and hemolytic anemias, and chronic kidney disease due to other and unspecified causes were also common causes of anemia in older adults aged 60 and above. The etiology of anemia in older age is complex and ranges from bone marrow failure syndromes to chronic kidney disease, and from nutritional deficiencies to inflammatory processes (16). Iron Deficiency Anemia (IDA) is the most common nutritional deficiency disorder (53). Although it has primarily been regarded as a public health concern affecting growing children, premenopausal women, and pregnant females, it is increasingly recognized as a clinical condition that can affect patients presenting to various medical and surgical specialties, especially those with chronic conditions and the older adults (54). Thalassemia is a recessive monogenic hematological disorder, which accounts for the largest share of this burden. Previous studies have indicated that it affects around 10% of the population in certain southern regions of China (55). A related study showed that hemoglobinopathies accounted for 20% of all anemia cases, ranking second in all cause-specific anemia in China (56). As individuals age, the older adults population often experiences multiple chronic conditions. One of the significant complications of chronic kidney disease, for example, is anemia (57). The analysis of the causes of anemia is essential to prevent and control anemia and reduce the burden of anemia in older adults. For example, iron deficiency can be controlled through iron supplementation, and iron fortification is an effective strategy to control dietary iron deficiency (58). China has the highest number of thalassemia cases in the world, prenatal screening can reduce the incidence of thalassemia (59).

Additionally, to reduce the burden of anemia in older adults aged 60 and above, it is crucial to monitor and prevent the onset and progression of other hemoglobinopathies hemolytic anemias, and chronic kidney disease due to other and unspecified causes. Furthermore, due to the varying causes of anemia across different age groups, the focus of concern among older adults individuals also differs by age group. For older adults aged 60–69, the focus should be on dietary iron deficiency, thalassemia traits, other hemoglobinopathies, and hemolytic anemias. In the 70–74 age group, 75–79 age group, males in the 80–84 age group, and those aged ≥85, the focus should shift to dietary iron deficiency, thalassemia traits, and chronic kidney disease due to other and unspecified causes. For females aged 80–85, attention should be given to iron deficiency, chronic kidney disease due to other and unspecified causes, and other hemoglobinopathies and hemolytic anemias.

To effectively reduce the anemia burden among China’s older adults population, targeted strategies should focus on high-risk groups and dietary optimization. First, priority should be given to vulnerable subgroups, including females, rural areas, western region, southern China, and the high-age older adults. Second, the causes of anemia in the older adults were complex. Dietary iron deficiency was the leading cause and one that can be directly and effectively controlled to prevent and treat anemia in older adults. Therefore, older adults should pay attention to proper dietary iron in their daily diet. At the same time, because the absorption and metabolism of different foods and their nutrients may interact and have a complex, cumulative effect on the body, it is also necessary to consider the comprehensive effect between various foods and nutrients to achieve nutritional balance.

Several strengths were exhibited in our study. Firstly, three large and nationally representative surveys were used to obtain data on the prevalence of anemia, including the 2002 CNHS, the 2010–2013 CNHS, and the 2015 CACDNS. In addition, part of the data was obtained from the public database GBD 2021, which incorporates surveillance and survey data to provide a comprehensive overview of trends and the current burden of anemia among the older Chinese population. However, several limitations warrant attention. First, cross-sectional studies, while valuable for identifying associations, have inherent limitations when it comes to making causal inferences. These studies provide a snapshot of relationships at a single point in time, which makes it difficult to establish temporal sequences or causal direction, and the real burden of anemia in the older adults may be ignored during the interval between the three surveys. So large cohort studies are needed to determine causality in the future. Second, while various data sources were integrated by the GBD 2021 study to ensure accuracy, deviations from the actual situation were unavoidable because the data were fitted by the models. While this study provided a comprehensive assessment of anemia burden and etiology, it did not explore multivariate interactions among risk factors (e.g., effect modification by socioeconomic status). Therefore, future studies are need to analyze the relationship with risk factors and anemia.

## 5 Conclusion

In 2002, 2010–2013, and 2015, the prevalence of anemia among older adults aged 60 and above, showed a decreasing trend. Meanwhile, in 2002, 2010, 2015, and 2021, YLD rates caused by anemia also demonstrated a downward trend over time. However, age and regional differences in the burden of anemia disease are still worth attention. Additionally, the prevalence, YLDs, and YLD rates of anemia increased with age. China is experiencing population aging, which indicates that anemia among older adults aged 60 and above is a common health concern. Moreover, greater attention should be paid to anemia in rural and western regions. Dietary iron deficiency is the leading cause of anemia across all age groups and both genders, highlighting the need for targeted interventions to address this nutritional deficiency.

## Data Availability

The datasets presented in this article are not readily available because the datasets from the 2002 CNHS, 2010 CNHS, and 2015 CACDNS used and/or analyzed during the current study are not publicly available according to the policy of the National Institute for Nutrition and Health, Chinese Center for Disease Control and Prevention. The data from the GBD 2021 is open to the public and can be obtained free of charge through website. Requests to access the datasets should be directed to https://vizhub.healthdata.org/gbd-results/.
